# Neurostimulation stabilizes spiking neural networks by disrupting seizure-like oscillatory transitions

**DOI:** 10.1038/s41598-020-72335-6

**Published:** 2020-09-21

**Authors:** Scott Rich, Axel Hutt, Frances K. Skinner, Taufik A. Valiante, Jérémie Lefebvre

**Affiliations:** 1 Division of Clinical and Computational Neuroscience, Krembil Research Institute, Toronto, ON Canada; 2 Team MIMESIS, INRIA Nancy Grand Est, Strasbourg, France; 3 Departments of Medicine (Neurology) and Physiology, University of Toronto, Toronto, ON Canada; 4 University of Toronto, Institute of Biomaterials and Biomedical Engineering, Toronto, ON Canada; 5 University of Toronto, Institute of Medical Science, Toronto, ON Canada; 6 Department of Surgery, Division of Neurosurgery, University of Toronto, Toronto, ON Canada; 7 Electrical and Computer Engineering, University of Toronto, Toronto, ON Canada; 8 Department of Biology, University of Ottawa, Ottawa, ON Canada; 9 Department of Mathematics, University of Toronto, Toronto, ON Canada

**Keywords:** Network models, Computational neuroscience, Dynamical systems, Epilepsy

## Abstract

An improved understanding of the mechanisms underlying neuromodulatory approaches to mitigate seizure onset is needed to identify clinical targets for the treatment of epilepsy. Using a Wilson–Cowan-motivated network of inhibitory and excitatory populations, we examined the role played by intrinsic and extrinsic stimuli on the network’s predisposition to sudden transitions into oscillatory dynamics, similar to the transition to the seizure state. Our joint computational and mathematical analyses revealed that such stimuli, be they noisy or periodic in nature, exert a stabilizing influence on network responses, disrupting the development of such oscillations. Based on a combination of numerical simulations and mean-field analyses, our results suggest that high variance and/or high frequency stimulation waveforms can prevent multi-stability, a mathematical harbinger of sudden changes in network dynamics. By tuning the neurons’ responses to input, stimuli stabilize network dynamics away from these transitions. Furthermore, our research shows that such stabilization of neural activity occurs through a selective recruitment of inhibitory cells, providing a theoretical undergird for the known key role these cells play in both the healthy and diseased brain. Taken together, these findings provide new vistas on neuromodulatory approaches to stabilize neural microcircuit activity.

## Introduction

Epilepsy is a condition in which the brain paroxysmally and repeatedly transitions into a seizure, a short lived state characterized by excessive neural activity, varying degrees of exaggerated synchronization and desynchronization, and oscillatory dynamics^1,2^. Medication is the first clinical option for patients with epilepsy, although when not effective such individuals are deemed to be medically refractory. Although half of medically refractory patients are not candidates for resective surgery, they may benefit from the use of neuromodulatory devices^3–7^. Neuromodulation in epilepsy is used to prevent the transition to seizure, or extinguish pathological oscillatory activity associated with seizures^8^. Current approaches seek to reduce seizures using either chronic open loop stimulation to alter circuit excitability^9^ (i.e. vagus nerve stimulation^10^), or closed-loop stimulation where seizures are detected in real time and electrical stimulation delivered in an attempt to abort an imminent seizure^11–14^. To improve the efficacy of neuromodulation for epilepsy, it is important to understand how such electrical stimulation impacts recurrent neural circuits^15–18^, particularly how neuromodulation may make these circuits more resilient to the dynamics characterizing seizures.

Experiments using optogenetic tools to selectively activate and inhibit specific cell-types are starting to reveal cell-type specific roles in seizure initiation and propagation. In contradistinction to these cell-type specific experiments that can be performed *in vitro* and *in vivo* in transgenic animals, the clinical application of neuromodulation is currently limited to the non-specific delivery of electrical current to the brain. While the real-world clinical benefits of neuromodulation have been demonstrated both for epilepsy^3–7^ and for movement disorders using deep brain stimulation (DBS)^16,19,20^, our understanding of how these effects are realized in neuronal circuits remains speculative at best^17,18^. In this context it has been opined that “neurostimulation for epilepsy often has moved retrograde, from pilot clinical studies back to the laboratory for validation and modification of stimulation methods”^21^.

In this vein we sought to understand the effects of neuromodulation on seizure-like transitions from a computational and mathematical perspective, allowing a principled understanding of dynamical interactions between modeled neurons and stimulus parameters. There are numerous existing computational models of ictogenesis^9,22–25^ and seizure propagation^26–28^. Irrespective of the different neurophysiological mechanisms involved in ictogenesis^29^, these in silico models generally represent seizure onset via spontaneous transitions between irregular spiking dynamics and hyperactive oscillatory states. Mathematically speaking, analogous transitions typically occur via passage through a cascade of dynamical instabilities called bifurcations^30^, mediated by an ad hoc control parameter (often via a parameter broadly encapsulating “neuronal excitability”). Such systems tend to be non-linear, meaning that the relationship between the inputs to the system and the outputted dynamics is not always proportional. These non-linear features are generic and essential ingredients of bifurcations and play a fundamental role in seizure-like state transitions in these models^24,31^; in fact, without non-linearities, many existing models lose the ability to exhibit these state transitions. Such non-linearities interact with noise to give rise to a plethora of noise-induced effects^32–35^.

Given the very different roles that cell-types have in inctogenesis^27,36–52^, we chose a simplified yet fully mathematically tractable model built of both excitatory and inhibitory cells with non-linear recurrent connections. Such two-population models have a long history of applications in neuroscience and brain modeling^53,54^ and generically display stereotyped dynamics such as synchronous oscillatory dynamics and multi-stability, which are core features observed in many seizure models^55^. The relative simplicity and analytical tractability of such models makes them an ideal candidate to study how neuromodulatory approaches engage recurrent cortical microcircuits in a theoretical setting, and similarly how stimulation interferes with the machinery involved in ictogenesis.

Using this framework, we explored how seizure-like sudden transitions into oscillatory activity are influenced by both intrinsic and extrinsic inputs, where these extrinsic inputs can be viewed as a model of a neuromodulatory intervention. Building on previous theoretical results^33,55–58^, our analysis demonstrates that stimulation, be it noisy or periodic in time, suppresses non-linear state transitions by preferentially recruiting the activity of inhibitory neurons without otherwise significantly altering the system’s dynamics. We provide mathematical evidence for the viability of this mechanism and show that extrinsic stimulation can be tuned to optimize this stabilizing effect. We note that a similar dynamic, a bistability in a purely inhibitory network, provides a mechanism underlying the increased interneuronal activity observed immediately prior to seizure onset in recent work^45^. Viewed together with this previous work, our results support a general hypothesis that stabilization of neural network dynamics away from sudden state transitions reduce their susceptibility to dynamics associated with the transition into seizure.

Our in silico results suggest that the activity of inhibitory interneurons, in particular their response to both intrinsic and extrinsic noise, acts to stabilize neural activity away from a seizure-like state in vivo. We additionally extend these results to other periodic stimulation waveforms to show that high-frequency stimuli of different types can also be used to generate the same effect. Taken together, this research provides mathematical support for further investigation into an expanded variety of neuromodulatory paradigms with the potential to increase a neural network’s resilience to seizure.

## Methods

### Oscillatory network of spiking excitatory and inhibitory cells

Since the focus of this work is primarily the interplay between excitatory and inhibitory populations, rather than the dynamics of individual cells, we use Wilson–Cowan models as opposed to other phenomenological or cellular-based models. Consider the following Wilson–Cowan-like network of excitatory and inhibitory populations ($u^e$ and $u^i$ respectively) subjected to recurrent connections and intrinsic noise^33,53,57^

1 $$a_e^{-1}\frac{d}{dt}u_j^e=L\left[ u_j^e\right] +N_e^{-1}\sum _{k=1}^{N_e}{w_{jk}^{ee}\ X_k^e(t)}+N_i^{-1}\sum _{k=1}^{N_i}{w_{jk}^{ie}\ X_k^i(t)}+I^e+I^o+\sqrt{2D}\xi _j^e\left( t\right) +S(t)$$

2 $$a_{i}^{{ - 1}} \frac{d}{{dt}}u_{j}^{i} = L\left[ {u_{j}^{i} } \right] + N_{e}^{{ - 1}} \sum\limits_{{k = 1}}^{{N_{e} }} {w_{{jk}}^{{ei}} X_{k}^{e} (t)} + N_{i}^{{ - 1}} \sum\limits_{{k = 1}}^{{N_{i} }} {w_{{jk}}^{{ii}} \;X_{k}^{i} (t)} + I^{i} + \sqrt {2D} \xi _{j}^{i} \left( t \right) + S\left( t \right)$$

with the linear operator $L[u]=-u$ and the membrane rate constants $a_e$ and $a_i$. The spike trains of excitatory and inhibitory cells $X_k^{e,i}(t)$ are governed by

3 $$\begin{aligned} X_k^{e,i}\left( t\right) =\sum _{{t_i}}{\delta (t-t_i)} \end{aligned}$$

where $t_i$ are the firing times of the cells whose firing follows a Poisson process with rate $f[u_k^{e,i}]$. The firing rate response function *f* is a sharp sigmoid of the form

4 $$\begin{aligned} f\left[ u\right] =\left( 1+\exp {\left( -\beta u\right) }\right) ^{-1}\ \end{aligned}$$

The constants $I^e$ and $I^i$ correspond to baseline currents to excitatory and inhibitory cells, respectively, and $I^o$ is an additional external excitatory input. The parameters $w_{jk}^{nm}$, $n,m=e,i$, are the connectivity weights between cells *j* and *k* of the populations *n* and *m*. Each cell is subjected to zero mean intrinsic Gaussian white noise $\xi _j^{e,i}(t)$, which is further uncorrelated in time and across cells, i.e. $<\xi _j^{e,i}(t)\xi _k^{e,i}(s)>=\delta (t-s)\delta (k-j)$, and *D* is the noise variance. The external stimulation *S*(*t*) is global, i.e. identical at each cell, and can take various forms (to be defined in the following). Parameters chosen here are listed in Table 1: these follow previous work of the authors in cortical networks made of excitatory and inhibitory populations^33,57^ and were chosen based on previous cortical modeling work in the literature^59–61^. An investigation regarding the robustness of our main findings to varying E/I balances can be found in the Supplemental Information.

**Table 1:** Model parameters.

Symbol	Definition	Value
$N_e$	Number of excitatory neurons	800
$N_i$	Number of inhibitory neurons	200
$\beta$	Response function gain	300
*h*	Response function threshold	0.0
$a_e$	Denritic rate constant, excitatory neurons	100 Hz
$a_i$	Denritic rate constant, inhibitory neurons	200 Hz
$w^{ee}$	Excitatory-excitatory synaptic connection strength	1.60
$w^{ei}$	Excitatory-inhibitory synaptic connection strength	3.00
$w^{ie}$	Inhibitory-excitatory synaptic connection strength	− 4.70
$w^{ii}$	Inhibitory-inhibitory synaptic connection strength	− 0.13
$I^e$	Baseline current, excitatory neurons	− 0.25
$I^i$	Baseline current, inhibitory neurons	− 0.50
$I^o$	Additional drive (i.e. stimulus) to excitatory neurons	Variable
*D*	Additive noise level	Variable
*dt*	Integration time step	1 ms

**Figure 1:**
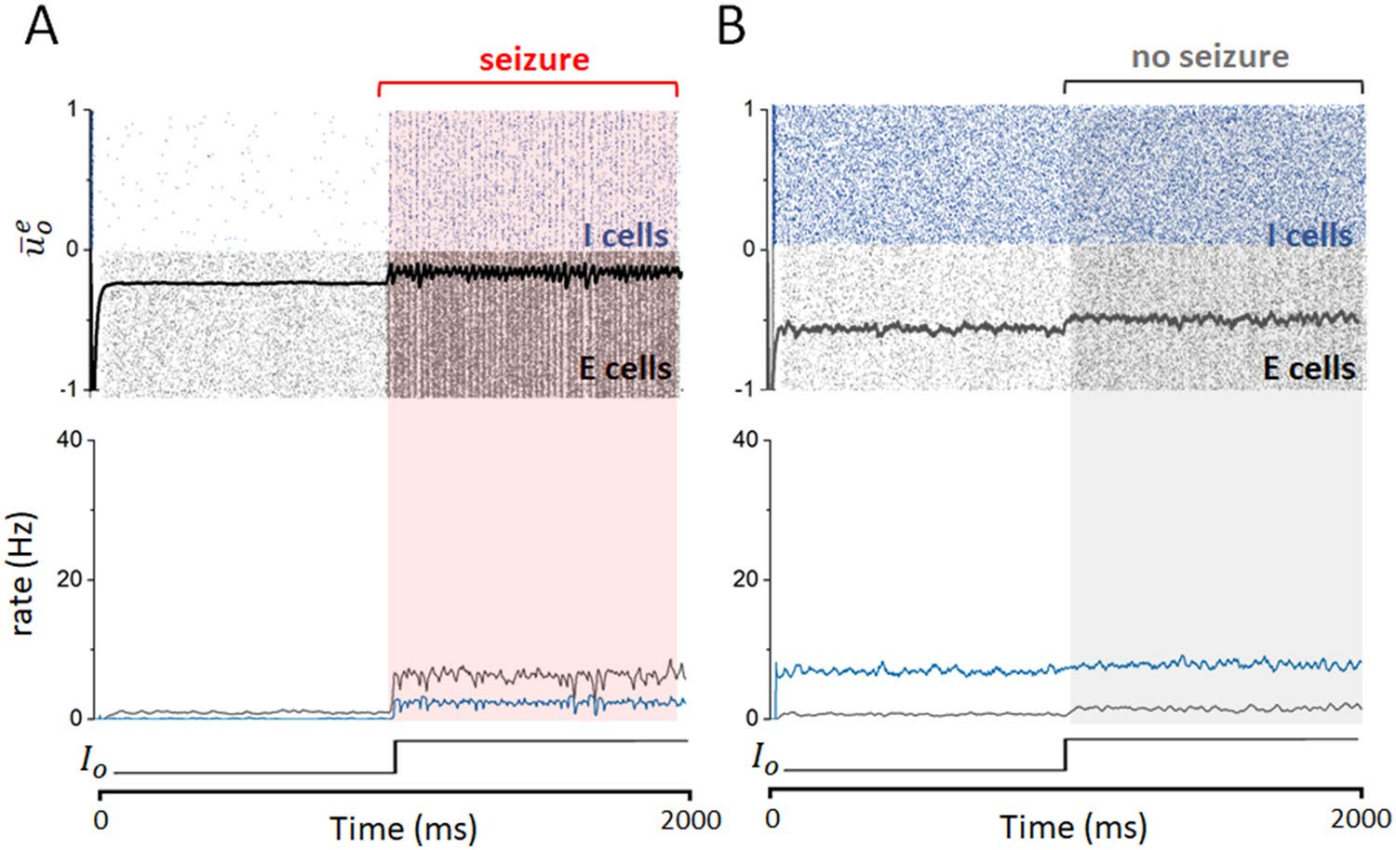
Noise-induced stabilization of network dynamics away from oscillatory activity. (**A**) An example simulation of the spiking network of excitatory and inhibitory cells (here with 10,000 cells, 8,000 excitatory and 2,000 inhibitory), with the intrinsic noise variance $D=0.01$. At T = 1,000 ms, excitatory input is abruptly increased from 0 to 0.1, leading to a sharp transition to oscillatory activity associated with synchronous neural firing. The mean membrane activity of the excitatory population exhibits strong periodic fluctuations with an approximate frequency in the high-beta/low-gamma range. This correlates with the spiking activity ($X^{e,i}(t)$ terms in Eqs. (1) and (2)) represented in a raster-plot format (blue dots indicate inhibitory cell spikes, and black dots are excitatory cell spikes) overlaid on this plot (upper panel). The firing rates of both excitatory (black curve) and inhibitory (blue curve) cells undergo a sudden jump while neuronal firing becomes maximally coherent (lower panel). (**B**) Under analogous conditions with higher intrinsic noise ($D=0.05$, again 10,00 cells), oscillatory activity is not observed. Spiking activity remains asynchronous and irregular throughout, and mean membrane activity is exempt of characteristic large scale periodic fluctuations (upper panel). The firing rate of the inhibitory population increases slightly following the increase in external drive, with minimal change observed for excitatory cells, distinct from what is seen in panel (**A**) (lower panel).

For the set of parameters chosen (see Table 1), the network generically displays input-driven shifts between asynchronous dynamics and oscillatory activity also consisting of synchronous neuron firing. Representative network activity is plotted in Fig. 1A, both before ($I^o=0$) and after ($I^o=0.1$) tonic excitation of the excitatory cells. There are parallels between these transitions and seizure onset, considering seizure onset is similarly sudden and seizure dynamics are typically oscillatory in nature (more specifically, seizures can be viewed as a type of oscillatory activity, although not all oscillations are seizure-like). Considering the similarities between the resulting dynamics and some features of seizure-like activity observed experimentally^1,48–50^, we study these transitions as theoretical approximations of seizure onset. A bifurcation analysis of this system is straightforward, and highlights not only the influence of network parameters on resulting dynamics, but also how different forms of stimulation might prevent sudden state transitions in the network.

We note two potential biophysical interpretations of the $I^o$ term initiating the seizure-like behavior of our model. Indeed, while there is consensus that epilepsy is a dynamical disease in which a neural network evolves as an “ictal attractor”^62,63^, there is no consensus as to how the system transitions into this attractor and the corresponding seizure state. One hypothesis is that an underlying neuromodulatory mechanism may cause this transition by inducing increased excitatory drive to the ictogenic zone^62^. Another is found in reflex seizures, where a sensory input represents the trigger initiating seizure^64^ if the stimulus statistics support synchronous neural activity^65,66^. In either interpretation, the $I^o$ term is related to biophysical processes that activate specific neural populations resulting in increased input to the seizure onset zone. This term can also be viewed as analogous to the “slow” or “control” parameters that trigger the transition into seizure in other seizure models (see for example the *z* slow variable in the Epileptor^26^).

### Stochastic mean-field reduction

We here follow the lines of previous work^55,56^ while extending it to two population spiking networks^57^. We assume that the firing rate of cells is sufficiently high (i.e. the population firing rate $N_{e,i}f$ is larger than the membrane rate $a_{e,i}$) to make use of the diffusion approximation^67^ appropriate, and gain

5 $$\begin{aligned} X_k^{e,i}\approx \ f[u_k^{e,i}] \end{aligned}$$

to obtain the self-consistent set of non-linear stochastic differential equations

6 $$\begin{aligned} a_e^{-1}\frac{d}{dt}u_j^e= & {} L\left[ u_j^e\right] +N_e^{-1}\sum _{k=1}^{N_e} w_{jk}^{ee}f[u_k^e]+N_i^{-1}\sum _{k=1}^{N_i}w_{jk}^{ie}f[u_k^i]+I^e+I^o+2D\xi _j^e(t)+S(t) \end{aligned}$$

7 $$\begin{aligned} a_i^{-1}\frac{d}{dt}u_j^i= & {} L\left[ u_j^i\right] +N_e^{-1}\sum _{k=1}^{N_e} w_{jk}^{ei}f[u_k^e]+N_i^{-1}\sum _{k=1}^{N_i}w_{jk}^{ii}f[u_k^i]+I^i+2D\xi _j^i(t)+S(t) \end{aligned}$$

We also introduce the mean membrane activities $\bar{u}^{e,i}$

8 $$\begin{aligned} {\bar{u}}^{e,i}(t)={\frac{1}{{TN}_{e,i}}}\int _{t}^{t+T} \sum _{k=1}^{N_{e,i}}{u^{e,i}\left( t^\prime \right) \ dt^\prime } \end{aligned}$$

with average period *T* that is much shorter than the time scale of the dynamics of the population mean. We further assume that the system primarily evolves in the mean-driven regime and local activity obeys

9 $$\begin{aligned} u_j^{e,i}\left( t\right) ={\bar{u}}^{e,i}\left( t\right) +v_j^{e,i}(t) \end{aligned}$$

where deviations $v_j^{e,i}$ are small. Then the mean activities for excitatory and inhibitory populations obey^57^

10 $$\begin{aligned} a_e^{-1}\frac{d}{dt}{\bar{u}}^e= & {} L\left[ {\bar{u}}^e\right] +{\bar{w}}^{ee}\ F_e\left[ {\bar{u}}^e\right] +{\bar{w}}^{ie}F_i\left[ {\bar{u}}^i\right] +I^e+I^o+\mu _S \end{aligned}$$

11 $$\begin{aligned} a_i^{-1} \frac{d}{dt}{\bar{u}}^i= & {} L\left[ {\bar{u}}^i\right] +{\bar{w}}^{ei}\ F_e\left[ {\bar{u}}^e\right] +{\bar{w}}^{ii}F_i\left[ {\bar{u}}^i\right] +I^i+\mu _S \end{aligned}$$

where $\bar{w}^{nm}=\sum _{k=1}^{N_n}\sum _{j=1}^{N_m}w_{kj}^{nm}/N_nN_m$, $\mu _s=\frac{1}{t}\int _t^{t+T}S(t')dt'$ and the effective transfer function

12 $$\begin{aligned} F_{e,i}\left[ u\right] =\ \int _{\Omega (v)}{f\left[ u+v\right] \rho ^{e,i}(v)dv} \end{aligned}$$

over some domain $\Omega (v)$ of deviations which is determined by the stimulation waveform. Indeed, the deviations from the mean $v_j^{e,i}$ follow a waveform-dependent probability distribution $\rho ^{e,i}(v)$^57,58^. This holds if $\bar{u}^{e,i}$ evolves on a time scale that is much larger than the average period *T*. Under this condition, and given an extrinsic stimulus *S*(*t*), deviations from the mean obey

13 $$\begin{aligned} {a_{e,i}}^{-1}\frac{d}{dt}v_j^{e,i} = L\left[ v^{e,i}\right] +\sqrt{2D}\xi _j^{e,i}+S(t)-\mu _s\ ~. \end{aligned}$$

Without stimulation, i.e. $S(t)=0$ (see below for the various cases with stimulation), the network dynamics are fully characterized by its parameters and intrinsic noise statistics. In this case, local deviations from the mean $v^{e,i}_j$ obey the independent and identically distributed (IID) Ornstein–Uhlenbeck processes

14 $$\begin{aligned} {a_{e,i}}^{-1}\frac{d}{dt}v_j^{e,i}=L\left[ v_j^{e,i}\right] +\sqrt{2D}\ \xi _j^{e,i}(t) \end{aligned}$$

and thus $\rho ^{e,i}(v)=N(0,a_{e,i}D)$, where *N* is the normal distribution with zero mean and variance $a_{e,i}D$.

### Stochastic stability analysis

We here follow the standard stability analysis of the Wilson–Cowan system^52,53,57^, but adapt it to account for the presence of additive noise. In the absence of stimulation, i.e. $S(t)=0$, a steady state $(\bar{u}_o^e,\bar{u}_o^i)$ is implicitly determined by

15 $$\begin{aligned} -L\left[ {\bar{u}}_o^e\right]\,=\, & {} {\bar{w}}^{ee}\ F_e\left[ {\bar{u}}_o^e\right] +{\bar{w}}^{ie}F_i\left[ {\bar{u}}_o^i\right] +I^e+I^o \end{aligned}$$

16 $$\begin{aligned} -L\left[ {\bar{u}}_o^i\right]\,=\, & {} {\bar{w}}^{ei}\ F_e\left[ {\bar{u}}_o^e\right] +{\bar{w}}^{ii}F_i\left[ {\bar{u}}_o^i\right] +I^i \end{aligned}$$

We note that intrinsic noise is still present here implicitly since the steady states of the network and their associated dynamics—are strongly impacted by the amplitude of intrinsic noise in the transfer function.

Linearizing about the steady state $(\bar{u}_o^e,\bar{u}_o^i)$ one obtains the linear system

17 $$\begin{aligned} \frac{d}{dt} \left( \begin{array}{c}a_e^{-1}\delta {\bar{u}}^e\\ a_i^{-1}\delta {\bar{u}}^i\\ \end{array}\right) =\mathbf{A} \left( \begin{array}{c}\delta {\bar{u}}^e\\ \delta {\bar{u}}^i\\ \end{array}\right) =\left( \begin{array}{cc} -1+{\bar{w}}^{ee}R^e&{}{\bar{w}}^{ie}R^i\\ {\bar{w}}^{ei}R^e&{}-1+{\bar{w}}^{ii}R^i\\ \end{array}\right) \left( \begin{array}{c}\delta {\bar{u}}^e\\ \delta {\bar{u}}^i\\ \end{array}\right) \end{aligned}$$

with $R^{e,i}=R(\bar{u}_o^e,\bar{u}_o^i)=\int _{\Omega (v)}f'[\bar{u}_o^{e,i}]\rho ^{e,i}(v)dv$. The stability of this system is given by the eigenvalues of matrix $\mathbf{A}$

18 $$\begin{aligned} \lambda _\pm =\frac{1}{2}G\pm \frac{1}{2}\sqrt{H}=\text {Re}\left[ \lambda \right] +i\text {Im}[\lambda ]=a+i\omega \end{aligned}$$

with

19 $$\begin{aligned} G\,=\, & {} {\bar{w}}^{ee}R^ea_e+{\bar{w}}^{ii}R^ia_i-\ (a_e+a_i\ ) \end{aligned}$$

20 $$\begin{aligned} H\,=\, & {} \left( {\bar{w}}^{ee}R^ea_e\right) ^2+\left( {\bar{w}}^{ii}R^ia_i\right) ^2-2 a_ea_iR^eR^i{\bar{w}}^{ee}{\bar{w}}^{ii}+4\ a_ea_iR^eR^i{\bar{w}}^{ei}{\bar{w}}^{ie}\nonumber \\&-2a_e^2R^e{\bar{w}}^{ee}+2a_ea_iR^e{\bar{w}}^{ee}+2a_ea_iR^e{\bar{w}}^{ii}-2a_i^2 R^i{\bar{w}}^{ii}-2a_ea_i+a_i^2+a_e^2 \end{aligned}$$

The damping rate (*a*) and eigenfrequency ($\omega$) of the system are the real and imaginary parts of the eigenvalues, respectively. The damping rate determines the stability of the steady state $(\bar{u}_o^e,\bar{u}_o^i)$ and allows us to investigate the role of model parameters and stimuli statistics in the population dynamics.

Notably, the influence of various parameters on the network stability can readily be examined. While our work focuses on the effect of stimulation, the analysis above is amenable to the examination of the role played by all parameters in dictating seizure-like behavior.

### Extrinsic noise stimulation

The case of extrinsic noise stimulation can be analyzed using a similar approach as intrinsic noise. In contrast to the case of intrinsic noise however, the external stimulus is global and both excitatory and inhibitory populations receive the same input. Hence it must be treated accordingly when deriving mean-field approximations and associated convolutions. Since realistic external stimuli do not have infinite power as white noise, their power spectral density has to decay for large frequencies. Consequently the noise stimulation *S*(*t*) is low pass-filtered Gaussian noise with $\mu _s=0$

21 $$\begin{aligned} \tau \frac{d}{dt}S=-S+\sqrt{2D_s}\eta (t) \end{aligned}$$

and with white noise $<\eta >=0$, $<\eta (t)\eta (t')>=\delta (t-t')$, time scale $\tau$ and noise variance $D_s$. The cut-off frequency of this low pass-filter is $f_c=1/\tau$, i.e. at this frequency the filter power corresponds to half its maximum value. Then the stationary probability density functions of $v_j^{e,i}$ (see Eq. 13) are $\rho ^{e,i}(v)=N(0, \bar{D}^{e,i})$ with effective noise variances

22 $$\begin{aligned} {\bar{D}}^{e,i}=a_{e,i}D+{a_{e,i}D}_sf_c/{(a}_{e,i}+f_c)\ . \end{aligned}$$

We see that the white noise-limit $f_c \rightarrow \infty$ yields the maximum effective noise strength $\bar{D}^{e,i} \rightarrow a_{e,i}(D+D_s)$ and retaining very low frequencies only reduces the impact of external stimulation since $\bar{D}^{e,i} \rightarrow a_{e,i}D$ for $f_c \rightarrow 0$.

Recalling that $f[u]=(1+\text {exp}(-\beta u))^{-1}$, if we set $\beta>>1$, then the firing rate function in Eq. (11) can be written explicitly as

23 $$\begin{aligned} F_{e,i}\left[ u\right] =\frac{1}{2}\left( 1+\text {erf}{\left[ -\frac{u}{2\left( {\bar{D}}^{e,i} \right) }\right] }\right) \end{aligned}$$

### Extrinsic periodic stimulation

Conversely to the extrinsic noise stimulation, periodic stimulation was analyzed numerically only. We refer the reader to our previous work^58^ for a more thorough mathematical analysis of the effects of the waveforms on the network response function.

#### Positive and negative pulse train stimulation

Pulse train stimuli correspond to successive, periodic and brief (1 ms duration, i.e. a single time step) excitatory and/or inhibitory inputs. The associated waveform is given mathematically by

24 $$\begin{aligned} S(t)=S_0\sum _n\delta (t-\frac{2\pi }{\omega _s}n) \end{aligned}$$

where $\delta (0)=1$ and zero otherwise. The pulse train has an angle frequency $\omega _s$ and an intensity of $S_0$. For $S_0<0$ and $S_0>0$ the stimulation is inhibitory and excitatory, respectively.

#### Biphasic pulse train stimulation

Biphasic pulse train stimuli combine excitatory and inhibitory pulse train stimuli, so they have a net zero mean. Brief, 1 ms pulses are alternated between positive and negative amplitudes. The associated waveform is given here by

25 $$\begin{aligned} S(t)=S_0\sum _n\delta \left( t-\frac{2\pi }{\omega _s}n\right) -S_0\sum _n \delta \left( t-\left( \frac{2\pi }{\omega _s}n + 2\Delta t\right) \right) \end{aligned}$$

where $\delta (0)=1$ and zero otherwise, and $\Delta t=1$ ms, our normal time step.

#### Sinusoidal stimulation

Extrinsic sinusoidal stimulation is a zero mean input whose waveform is given by

26 $$\begin{aligned} S(t)=S_0 \sin (2\pi \omega _s t) \end{aligned}$$

with angle frequency $\omega _s$ and amplitude $S_0$.

### Measure of neuromodulatory effectiveness

To quantify the impact of waveform parameters on state transitions into oscillatory activity, we simulated networks with stimuli of various amplitudes and frequencies (visualized using a heatmap) and calculated the spike coherence^68^. The larger the spike coherence in the population, the more synchronously the cells fire. A thorough investigation into the dynamics of this system revealed a clear correspondence between synchronous spiking and oscillatory network dynamics, and as such this spike coherence measure is used to quantify the network’s tendency to oscillate.

Spike coherence was measured after an increase in the tonic drive to the excitatory population $I^o$ that triggers ocillatory dynamics in the absence of neuromodulatory stimulus. The stimulation paradigm of interest was applied continuously both before and after this change in input. To do so, we randomly selected two excitatory cells *i*, *j* and binned their respective responses over a given time window $\Delta t$=20 ms. For cell *j*, $X_j^{\text {bin}}(k\Delta t)=1$ if a spike occurred during the interval $[k\Delta t, (k+1)\Delta t]$, and zero otherwise, where $k=0,\ldots ,T/\Delta t-1$ and *T* is the duration of the spike train. Spike coherence was then computed using

27 $$\begin{aligned} C\left( \Delta T\right) =\frac{\sum _{T}{X_i^{bin}X_j^{bin}}}{\sum _{T}\ X_i^{bin\ 2}\sum _{T}\ X_j^{bin\ 2}} \end{aligned}$$

where $\sum _T$ denotes the sum over time intervals. For $C(\Delta T)=0$ two spike trains in the excitatory population are independent and no seizure is present, whereas $C(\Delta T)=1$ reflects maximum coherence.

**Figure 2:**
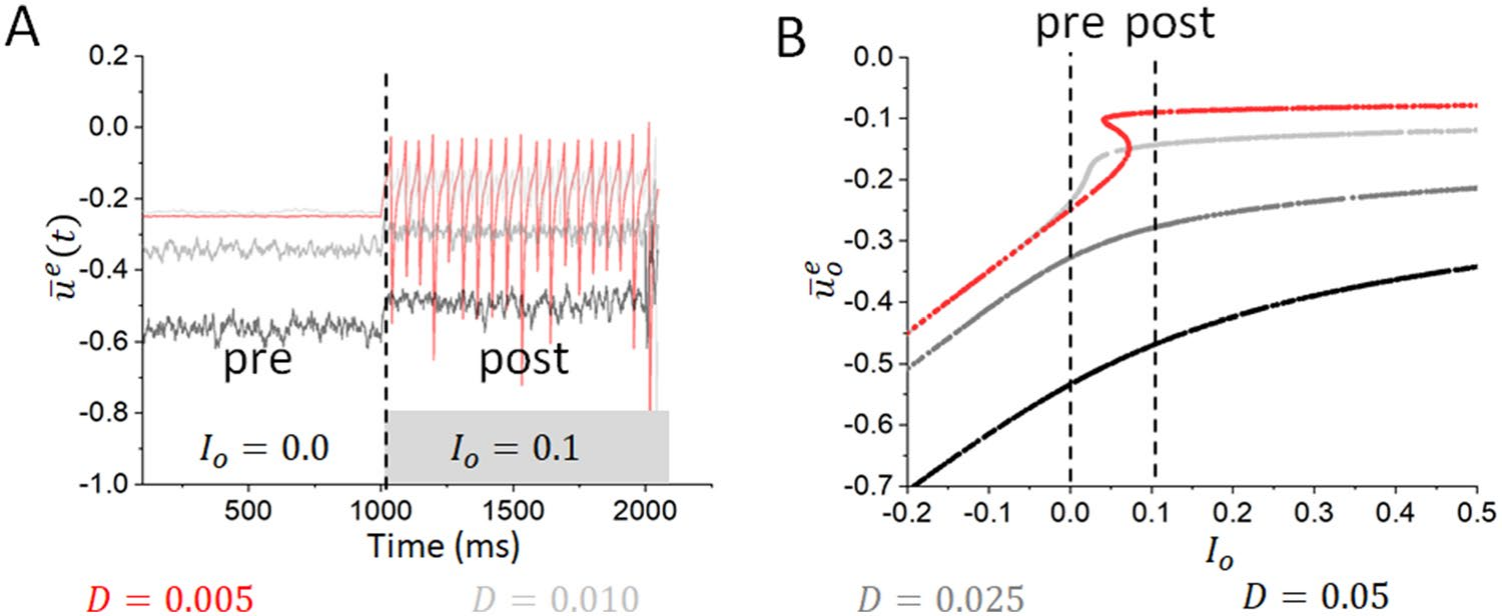
Multi-stability promotes transitions of the network into an oscillatory state. (**A**) The mean membrane activity of excitatory cells for different noise intensities (*D*), showing that dynamics become less oscillatory as the intrinsic noise variance increases. For the highest noise intensities ($D=0.05$ in black and $D=0.025$ in dark grey), the increase in the activity of excitatory cells in response to an increase in external drive is mild, whereas lower noise intensities ($D=0.005$ in red and $D=0.010$ in light grey) exhibit sudden and sharp transitions into oscillatory dynamics. Simulations here are with 10,000 cells. (**B**) The system exhibits multi-stability on a finite interval of excitatory drive for the lowest noise variance ($D=0.005$ in red), corresponding with the tendency to transition into oscillatory dynamics shown in panel A. In contrast, networks with higher intrinsic noise variances ($D=0.05$ in black and $D=0.025$ in dark grey) do not exhibit multi-stability, corresponding with the more continuous change in network dynamics seen in panel (**A**). $D=0.010$ (light grey) is an intermediate case where, while a obvious regime of multi-stability is not visible, there is an abrupt uptick in the value of the mean excitatory membrane activity ($\bar{u}^e_o$) corresponding with the increase in $I_o$ from 0 to 0.01 that is represented by the sudden increase in activity seen in panel (**A**).

**Figure 3:**
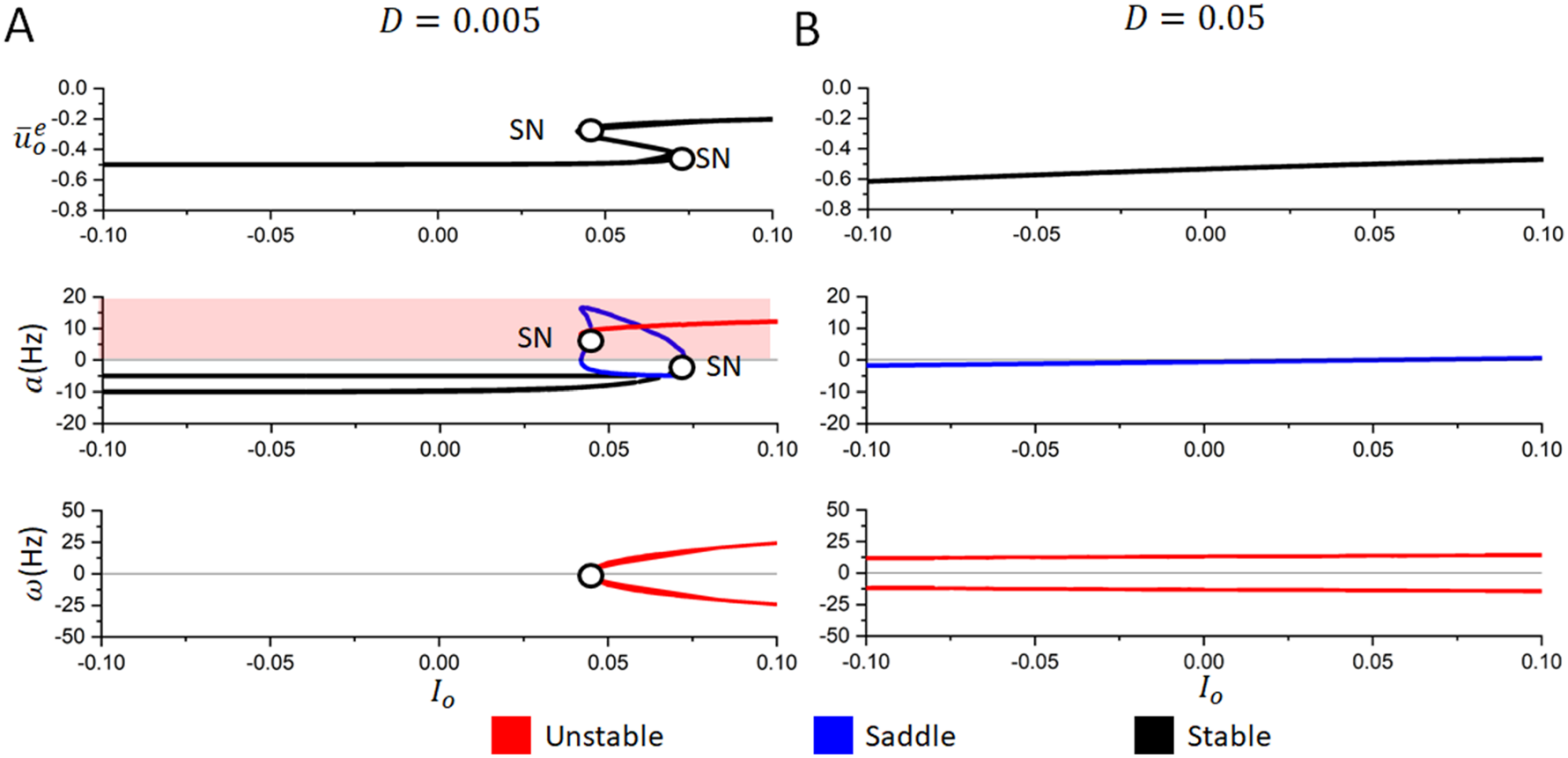
Stochastic stability analysis reveals effects of increased intrinsic noise. (**A**) With low intrinsic noise ($D=0.005$) the system is multi-stable for input currents ($I^o$) near 0.05, resulting in two saddle-node (SN) bifurcations. These bifurcations can be seen both in the plots of the stable values of the mean membrane potential of excitatory cells ($\bar{u}^e_o$, top graph) and in the real part of the associated eigenvalues (*a*, middle graph). Once this saddle node bifurcation occurs, the system transits towards a high-amplitude limit cycle, whose frequency ($\omega$, bottom graph) lies in the gamma range. (**B**) With higher intrinsic noise ($D=0.05$), multi-stability and bifurcations are not present, and stable equilibria are found for lower value of $\bar{u}^e_o$. The frequency of damped oscillation is here also much less than the one observed in the small-noise limit in panel (**A**).

We note that in the heatmap visualizations in Figs. 5 and 6, the stimulation frequencies on the x-axis are not uniformly spaced and do not exceed 200 Hz. This was necessary since in order to to maintain computational efficiency, our model resolved differences between stimulation paradigms whose periods were greater than 4 Hz and differed by at least 1 Hz. Thus, we simulated stimuli with periods of [1000, 500, 300:25:100, 90:10:50, 45:5:30, 28:2:20, 19:1:5] ms to reasonably sample a range of frequencies between 1 and 200 Hz.

### Mean firing rate

The mean firing rate of a given population over a short time period $\Delta T$ is given by the mean of the spiking activity over the population:

28 $$\begin{aligned} r^{e,i}(t) = {N^{e,i}}^{-1}\sum _{j=1}^{\Delta T^{-1} N^{e,i}}\int _t^{t+\Delta T} {X_j^{e,i}(s) ds} \end{aligned}$$

### Single cell power spectrum analysis

Excitatory and inhibitory cells have different intrinsic time scales^61^ and hence respond to external stimuli differently. For a decoupled cell, Eqs. (1) and (2) read

29 $$\begin{aligned} \frac{1}{a_{e,i}}\frac{du^{e,i}_i}{dt}=-u^{e,i}_i+\eta _i(t)~. \end{aligned}$$

The external input $\eta _i(t)=\xi _i(t)+S(t)$ is a linear superposition of the intrinsic noise $\xi _i(t)$ at node *i* and the extrinsic global stimulation *S*(*t*). For a time series of duration *T*, the power spectral density of $u^{e,i}_i$ is

30 $$\begin{aligned} S^2_{e,i}(\omega _n)= & {} \lim _{T\rightarrow \infty }\frac{a_{e,i}^2}{a_{e,i}^2+\omega _n^2} \left( D+|\tilde{S}_n|^2\right) ~. \end{aligned}$$

with discrete angle frequency $\omega _n=2\pi n/T$ and integer $n\in \mathbb {N}_0$. Here, we consider the intrinsic white noise process $\xi _i(t)$ with variance *D* that is uncorrelated to the extrinsic stimulation input *S*(*t*) and $\tilde{S}_n$ is the discrete Fourier transform of the global stimulus

31 $$\begin{aligned} \tilde{S}_n=\frac{1}{\sqrt{T}}\int _{-T/2}^{T/2} S(t)e^{-i\omega _nt}dt~. \end{aligned}$$

Equation (29) describes a lowpass-filter with input $\eta _i$. It has a cut-off angle frequency $\omega _c=a_{e,i}$ defined by $S^2_{e,i}(\omega _c)/S^2_{e,i}(0)=1/2$ (see Eq. 30). This means that input with angle frequency $\omega <\omega _c$ is said to pass the filter, whereas input with angle frequency $\omega >\omega _c$ is filtered out. This cut-off frequency is larger for inhibitory than for excitatory cells due to $a_i>a_e$ and hence $S^2_i(\omega )>S^2_e(\omega )$. Since this time scale separation and hence the different cut-off frequencies reflect physiological properties, inhibitory cells respond stronger to external stimuli than excitatory cells. However, we point out that the neural populations in our network are coupled and highly nonlinear. Thus, while this reasoning does not apply directly to the network but to individual cells, and as such additional coupling-induced effects should be expected in network dynamics, it may still provide insight into a potential mechanistic explanation for this result.

More specifically, the power spectra of

- the extrinsic noise stimulation *S*(*t*) with $\langle S(t) \rangle =0,~\langle S(t)S(t-\tau )\rangle =D_s\delta (\tau )$, $\langle \cdot \rangle =\int _{-T/2}^{T/2}\cdot dt/T$ with the $\delta$-distribution $\delta (\cdot )$ reads
  32 $$\begin{aligned} S^2_{e,i}(\omega _n)= & {} \frac{a_{e,i}^2\left( D+D_s\right) }{a_{e,i}^2+\omega _n^2}~. \end{aligned}$$
- the positive and negative pulse train stimulation, cf. Equation (24), now described by $S(t)=S_0\sum _{l}\delta (t-t_l),~S_0\in \mathbb {R}$, reads
  33 $$\begin{aligned} S^2_{e,i}(\omega _n)= & {} \lim _{T\rightarrow \infty }\frac{a_{e,i}^2}{a_{e,i}^2+\omega _n^2} \left( D+TS_0^2\left( \frac{\omega _s}{4\pi }\sum _{l=-N/2}^{N/2}(\delta _{n,lk} +\delta _{-n,lk})+\frac{1}{T}\right) ^2\right) \end{aligned}$$
  with $k=\omega _sT/2\pi$ and $t_l=2\pi l/\omega _s$ and *N* is the number of pulses in time *T*. Here we have applied the Poisson summation formula.
- the biphasic pulse train stimulation, cf. Equation (25), now described by $S(t)=S_0\sum _{l}(\delta (t-t_l)-\delta (t-t_l-\tau )),~S_0\in \mathbb {R}$ and $\tau >0,~\tau \rightarrow 0$, reads
  34 $$\begin{aligned} S^2_{e,i}(\omega _n)= & {} \lim _{T\rightarrow \infty }\frac{a_{e,i}^2}{a_{e,i}^2+\omega _n^2} \left( D+\frac{S_0^2\omega _s^2\tau ^2}{4T}n^2\sum _{l=-N/2}^{N/2}\left( \delta _{n,lk} +\delta _{-n,lk}\right) \right) ~. \end{aligned}$$
- the sinusoidal stimulus, cf. Equation (26), reads
  35 $$\begin{aligned} S^2_{e,i}(\omega _n)= & {} \lim _{T\rightarrow \infty }\frac{a_{e,i}^2}{a_{e,i}^2+\omega _n^2} \left( D+\frac{TS_0^2}{4}\left( \delta _{n,k}+\delta _{-n,k}\right) \right) ~. \end{aligned}$$

## Results

### Intrinsic noise, endogenous activity and susceptibility to state transitions

Given the chosen parameters (see Table 1; “Methods”), our model generically exhibits sudden shifts between asynchronous dynamics and oscillatory activity, typically including more synchronized neural spiking, mediated by increased excitatory input. Although our chosen network parameters and variables of interest may be “phenomenological” in nature^69^, the network exhibits the dynamical transition of interest that has important parallels with seizure onset. This assertion relies on the abrupt nature of this transition and the fact that seizure is itself an oscillatory state with specific features. Maintaining these phenomenological elements of the model allows for the necessary mathematical analysis to explore mechanisms of action for neuromodulation on these microcircuits with strong theoretical support.

Our model demonstrates a sharp transition to oscillatory dynamics (and corresponding synchronous neural firing) arising from relatively small increase in drive to the excitatory cells, as shown in Fig. 1. Oscillatory activity is common in networks with inter-connected excitatory and inhibitory cells, colloquially termed “E-I networks”^70–76^, making these sharp transitions from irregular activity into oscillatory dynamics expected for large portions of the parameter space. More importantly in the context of this study, and the mathematical exploration of this phenomenon, is that such behaviour is a signature of multi-stability: the existence of multiple stable solutions to a dynamical system. Indeed, such multi-stability represents a key feature of potential seizure-inducing mechanisms, often represented by a sudden transition into some form of oscillatory dynamics, both in vivo and in silico^45,55,62,77,78^. Both excitatory and inhibitory cells fire synchronously following the transition into oscillatory dynamics in our spiking networks (Fig. 1A). Motivated by these observations and building on previous findings on the interaction between noise and oscillatory activity^33,56,79^, we chose to further investigate the influence of intrinsic noise on the system’s predisposition to this multi-stability by examining the mathematical features necessary for bifurcations and seizure-like transitions to occur. We note that in Fig. 1A the frequency of the seizure-like oscillations are approximately in the high-beta/low-gamma range, paralleling the frequencies observed clinically in low voltage fast seizure onsets^80–83^.

We first numerically examined the influence of intrinsic noise on input driven oscillatory activity. Figure 1B demonstrates that increasing the intrinsic noise level (represented by the variable *D*) suppresses the transition from asynchronous dynamics into oscillatory activity. When subjected to an increase in excitatory drive, neural activity remained asynchronous and irregular with larger levels of intrinsic noise, with the network responding to the increase in this drive with only a small uptick in excitatory activity. In contrast, clear oscillatory dynamics are observed for the low values of *D* shown in Fig. 1A, corresponding with more substantial increases in excitatory activity. This suggests that intrinsic fluctuations driven by noise make the network’s response to increased external drive smooth, monotonic, and commensurate to the change in external drive. In Fig. 2A we show that this effect scales, i.e. that increases in the noise variance *D* gradually decrease the salience of the oscillatory activity (and correspondingly the non-linearity of the response to an increase in drive) until such oscillations are entirely absent.

This result may appear counter-intuitive, considering that the randomness inherent in noisy input may be expected to make a network’s dynamics less, rather than more, predictable. Detailed mathematical analysis is uniquely suited to uncover potential mechanisms explaining such results, especially in complex systems such as neural circuits. In this vein, we performed a stochastic mean field analysis (see “Methods”) on our network in search of a convincing explanation of this behavior. Fig 2B shows the steady state(s) of the system as a function of input drive (see “Methods”). For weak noise (i.e. low values of *D*), the system exhibits the multi-stable behaviour observed previously: the populations possess multiple stable states between which the system can transition abruptly due to shifts in the tonic excitatory drive. In the spiking network, these jumps are accompanied by a shift from irregular to oscillatory activity (as seen in Fig. 2A).

However, as illustrated by Fig. 2B, increasing the level of intrinsic noise gradually reduces the width of the multi-stable region in the parameter space until it vanishes completely. This corresponds with a decrease in oscillatory activity seen following an increase in the tonic excitatory drive in the spiking networks (Fig. 2A). Another important observation is the suppressing effect of noise on the level of activity in the excitatory resting states. Indeed, both within and outside the multi-stability region, increased intrinsic noise intensity shifts steady states to lower values of the mean potential. This indicates that, in the present model, noise serves to damp down excitatory activity. This is consistent with the noise-induced reduction in excitatory firing rates seen in Fig. 1.

**Figure 4:**
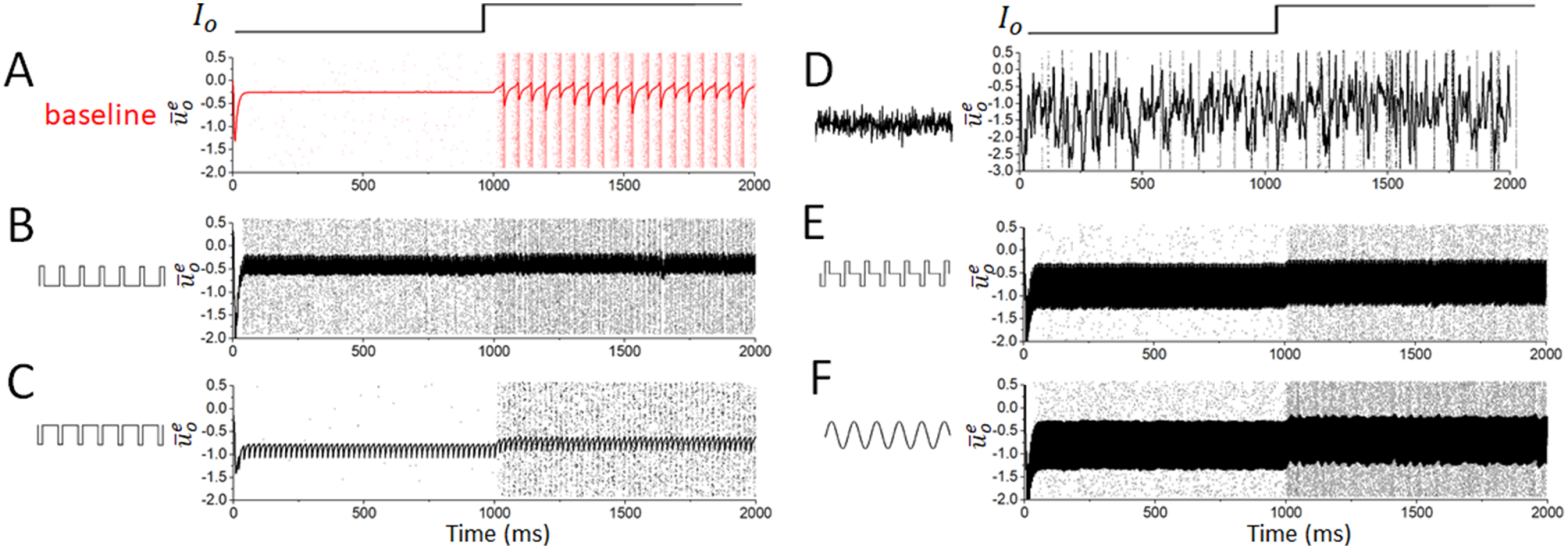
Various stimulation paradigms can all show stabilizing effects. (**A**) Example illustrating the transition of the baseline network into oscillatory dynamics following an increase in the external drive to the system. (**B**–**F**) Examples showing that each of the stimulation paradigms studied here (excitatory pulses with $S_o=1.5$ and $\omega _s=150$ Hz in panel (**B**), inhibitory pulses with $S_o=1.0$ and $\omega _s=50$ Hz in panel (**C**), noisy stimulation with $D_s=0.08$ and $f_c=400$ Hz in panel (**D**), biphasic stimulation with $S_o=2.5$ and $\omega _s=150$ Hz in panel (**E**) and sinusoidal stimulation with $S_o=2.5$ and $\omega _s=200$ Hz in panel (**F**)) can exert a stabilizing effect on the network away from the conspicuous oscillatory dynamics seen in panel (**A**). Instead, in each of these plots very similar behavior is observed both before and after the increased drive is applied.

Intrigued by the stabilizing role of intrinsic noise, we pushed our mean field analysis further and performed a linear stochastic stability analysis (see “Methods”). Figure 3 depicts the system’s steady state stability as a function of the excitatory tonic drive for two different noise variances, detailing how increases in input to the excitatory population impacts state transitions between asynchronous and oscillatory activity. For weak noise (Fig. 3A), increased drive leads to passage through the multi-stable states, in which the system undergoes a saddle node-homoclinic bifurcation^84^: increasing the excitatory drive causes the system to jump from a stable node to a remaining unstable oscillatory steady state and the population dynamics jump abruptly to a high-frequency limit cycle. In contrast, when intrinsic noise is increased in the system (Fig. 3B), the same change in excitatory drive does not cause such a transition: the unique steady state remains stable and at a lower level of the mean potential when compared to the results in Fig. 3A, conforming with the results of Fig. 2B.

The observations from our spiking network, confirmed by the use of the mathematical tool of stochastic stability analysis, provide a key insight: intrinsic noise stabilizes neural population activity by making responses to increases in external drive more commensurate to the degree of this change, i.e. the dynamics become more linear. In turn, this makes abrupt, major shifts in network dynamics (like those that occur during the sudden transition between asynchronous and oscillatory dynamics) unlikely. This well-known noise-driven phenomenon^56,58,79,85–87^ shapes the stability of this non-linear system.

With the effects of intrinsic noise on our neural microcircuit thoroughly explored, we asked whether similar stabilizing effects can be achieved by an extrinsic stimulus. Such a stimulus could be provided, in the in vivo setting, via a neuromodulatory device. We extend our mathematical and theoretical understanding from the intrinsic to the extrinsic setting in the following.

### Stabilizing network activity via external modulatory stimuli

Our analysis of the effects of intrinsic noise on this model microcircuit, combined with previous work, provides some intuition that extrinsic noise might have a similar stabilizing effect as intrinsic noise in this in silico setting. Indeed, high-frequency exogeneous inputs can be used to modulate endogenous neural oscillatory activity^58^, although it remains to be seen whether they can also be used to stabilize the system to prevent sudden dynamical transitions. We thus examined the role of various stimulation waveforms on the transition into oscillatory dynamics. Specifically, to test the effect of these waveforms on the multi-stability observed in our model, we suddenly increased the tonic drive to the excitatory population (which induces a transition between asynchronous and oscillatory dynamics in systems with low intrinsic noise in the absence of external modulation) while continuously (i.e. pre and post increased drive) subjecting the network to stimuli with various waveforms. Fig. 4 presents stimuli (see “Methods”) that exert a waveform-dependent effect on the oscillatory properties of our spiking networks. All five stimuli of interest can, for informed choices of the stimulus amplitude and frequency, exert a stabilizing effect on the network and prevent major changes in network dynamics following an increase in excitatory drive. We examine the differences between the effects of these waveforms in the following.

**Figure 5:**
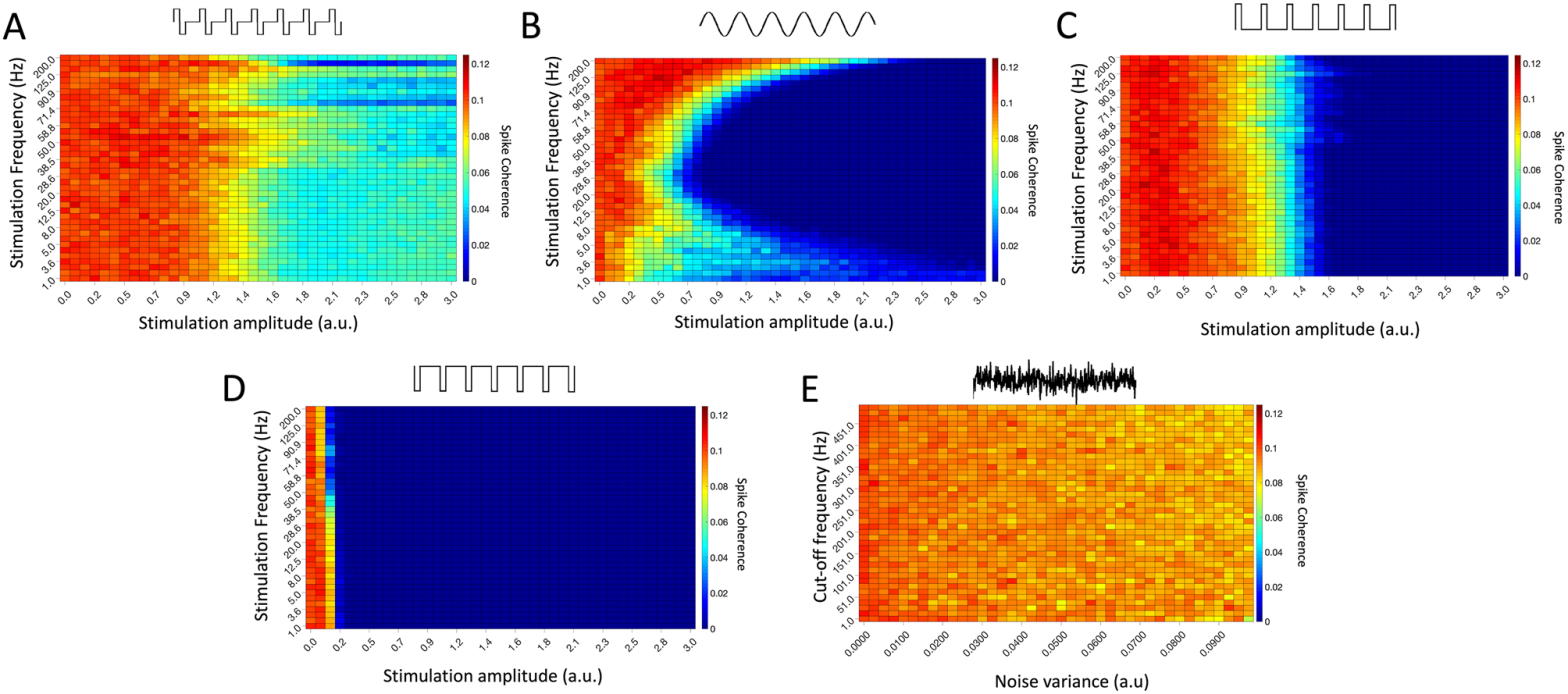
Stabilization of network dynamics by stimuli with different waveforms. (**A**–**D**) Spike coherence averaged over 10 independent simulations following an increase in excitatory drive for different neuromodulatory paradigms (biphasic stimuli in panel (**A**), sinusoidal stimuli in panel (**B**), excitatory pulses in panel (**C**) and inhibitory pulses in panel (**D**)). While different combinations of stimulation amplitude and frequency yield an optimal decrease in spike coherence for each waveform (as expected), generally each stimulus paradigm best minimizes network synchrony when the stimulus has high amplitude and frequency. (**E**) Spike coherence averaged over 10 independent simulations following an increase in excitatory drive for the noisy neuromodulatory paradigm. Here, coherence is diminished for stimuli with high noise variance and cut-off frequency, analogous to the high amplitude and frequency for other wave forms.

#### Response to extrinsic noise stimulation

Neuromodulation by noisy stimuli, or extrinsic noise, corresponds to the use of electric fields of irregular amplitudes and fluctuating frequencies to modulate the activity of superficial cortical neurons. Either non-invasive or intracranial, extrinsic noise stimulation is used in a variety of clinical settings to modulate cortical excitability^88^. It further has numerous and varied clinical applications, including reducing fatigue in multiple sclerosis patients^89^, improving creativity^90^ and alleviating tinnitus symptoms^91^. Its effect on ongoing alpha activity has been both computationally and mathematically characterized^58^.

We emphasize that, as discussed in detail in “Methods”, the primary difference between the intrinsic and extrinsic noise in this research is that the intrinsic noise is independently generated for each cell, while the extrinsic noise is uniform for each modeled cell.

Figure 4D shows an example of how extrinsic noise stimulation prevents a sudden shift into oscillatory dynamics. The effect of extrinsic noise on the tendency for the network to exhibit such dynamics following an increase in excitatory drive is further examined in Fig. 5E. Neuromodulation with the largest noise variances and cut-off frequencies (see “Methods”) yield diminished spike coherence following the seizure stimulus, indicating a tendency for less synchronous network dynamics, where network synchrony is used as a computationally efficient quantification of oscillatory behavior (see “Methods”). This implies that population synchrony can be suppressed, and sudden transitions into oscillatory activity mitigated, for multiple combinations of relatively high amplitudes and cut-off frequencies of this extrinsic noise stimulation.

**Figure 6:**
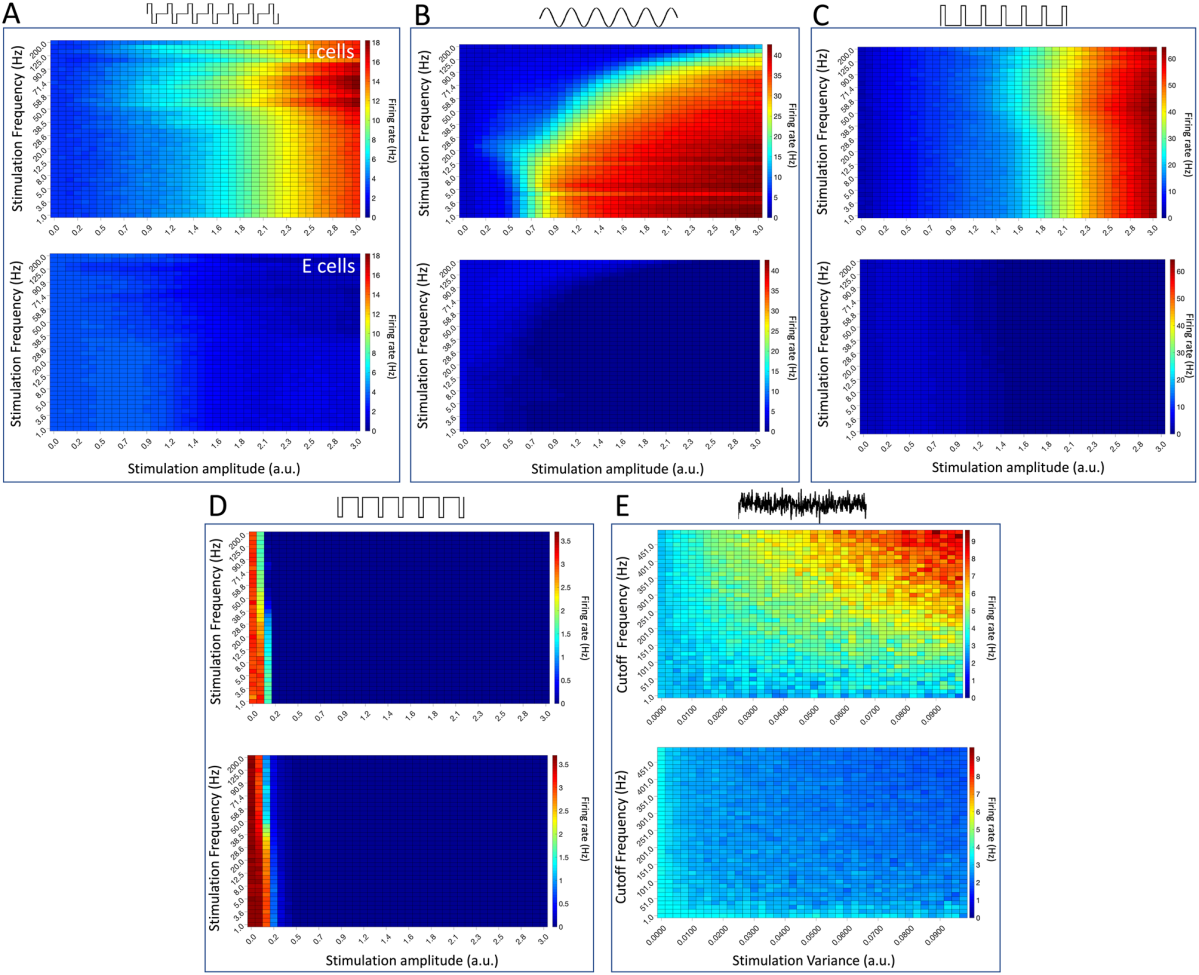
Firing rates of excitatory and inhibitory cells in response to stimulation with different waveforms for various stimulation parameters. (**A**–**E**) Firing rates for inhibitory (top) and excitatory (bottom) cells for the parameter ranges explored in Fig. 5. Biphasic stimulation is shown in panel (**A**), sinusoidal stimulation in panel (**B**), excitatory pulses in panel (**C**), inhibitory pulses in panel (**D**), and noisy stimulation in panel (**E**). Note that regimes of lowest spike coherence in Fig. 5 agree with regimes of enhanced inhibitory activity. Results are averaged over 10 independent simulations.

#### Response to periodic neurostimulation

As shown in earlier work^58^, stochastic stability analysis is amenable to high-frequency (non-stochastic) stimuli, whose effect can be examined and investigated using similar mean-field techniques. The influence of stimuli on ongoing neural activity depends on the statistical properties of their waveform, and can alter network dynamics by tuning its stability. These results suggest that high-frequency stimuli such as sinusoidal waves and pulse trains could interact with the neural populations and interfere with transitions into oscillatory dynamics.

Confirming this hypothesis, various non-noisy waveforms were found to disrupt the onset of oscillatory dynamics. We repeated the above analysis for inputs consisting of different waveforms, computing spike coherence in the network following the seizure stimulus as a function of stimulation amplitude and frequency. The results in Fig. 5A–D reveal a co-dependent role of amplitude and frequency in preventing oscillatory dynamics. Minimal spike coherence was achieved for high amplitude, high frequency stimuli for most waveforms. Different response patterns are expected given the waveform-dependent effect of these stimuli on the system response function^58^.

#### Differential excitatory and inhibitory responses to neurostimulation

To better understand the mechanism by which these stimulation paradigms, both noisy and periodic, decrease spike coherence and correspondingly disrupt the transition into oscillatory activity, we simultaneously computed the mean firing rates of excitatory and inhibitory cells during these explorations. The results plotted in Fig. 6 show that, under a wide variety of frequency and amplitude combinations, stimulation enables a differential recruitment of excitatory and inhibitory cells. Specifically, the simulations show that high-frequency, high-amplitude stimuli predominantly recruit inhibitory cells by increasing their firing rates, which in turn further suppresses excitatory activity. This occurs in the biphasic and positive pulsatile stimuli (Figs. 5A,C; 6A,C), in the sinusoidal stimuli (Figs. 5B, 6B) and in the noise stimulation (Figs. 5E, 6E). The negative pulsatile stimulus (Figs. 5D, 6D) is the lone exception, in which neuromodulation suppresses firing activity in both excitatory and inhibitory cells. In sum, stimulus-induced actions diminish excitatory neural firing and minimize spike coherence (Figs. 5, 6), showcasing a potential mechanism of action for the anti-ictogenic effects of neuromodulation through the selective activation of inhibitory cells.

**Figure 7:**
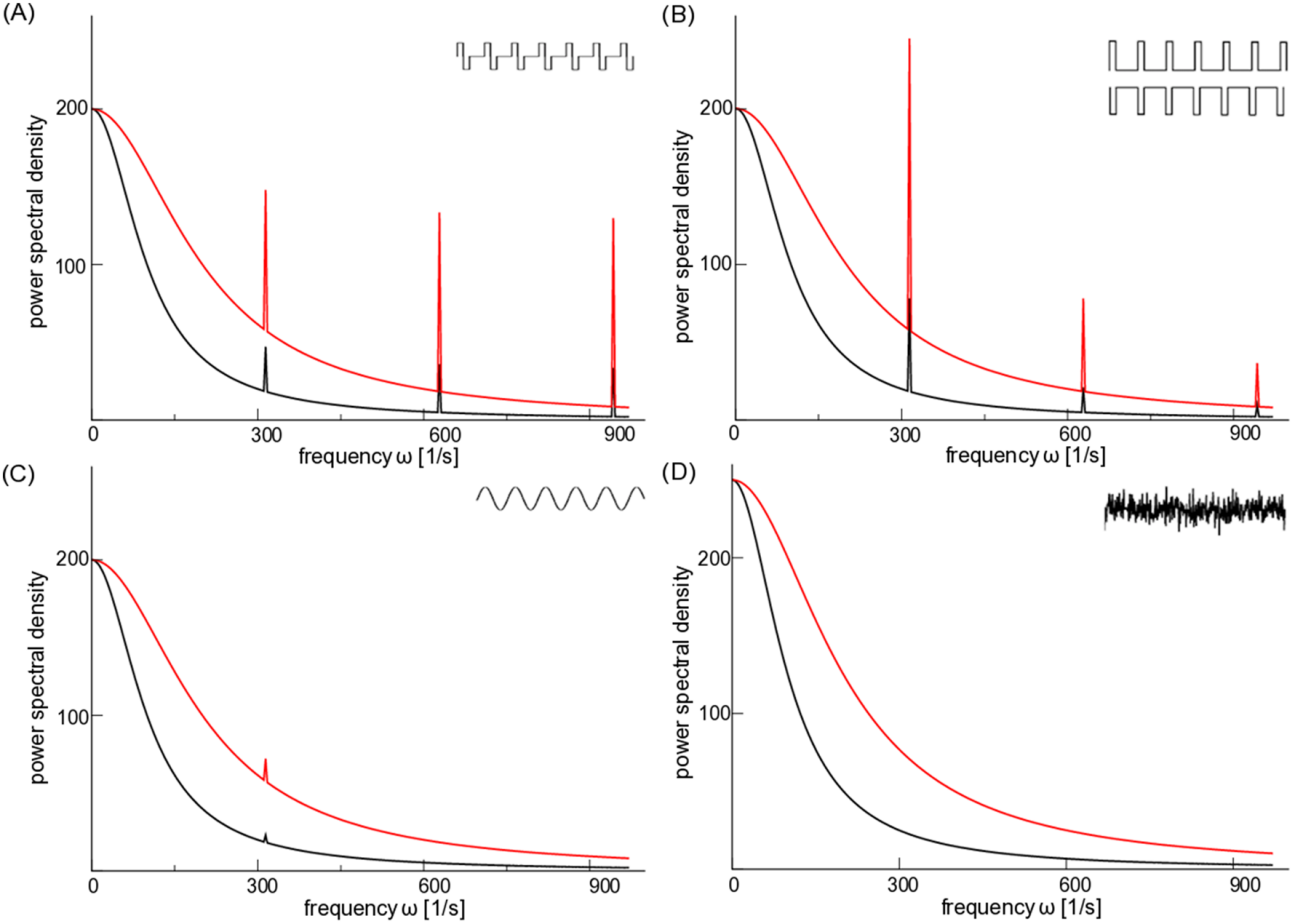
Power spectral density (PSD) of linearly responding excitatory and inhibitory cells. (**A**–**D**) The PSD of individual excitatory (black) and inhibitory (red) cells in response to four different stimuli (biphasic stimulation in panel (**A**), excitatory and inhibiory pulse trains in panel (**B**), sinusoidal stimulation in panel (**C**), and noisy stimulation in panel (**D**)). In all cases and for all frequencies measured, the power is higher for inhibitory than excitatory cells, potentially explaining their enhanced response to these neuromodulatory paradigms in our studied networks. Parameters are $S_0^2=0.5$, $a_e=100$Hz, $a_i=200$Hz, $D=200$, $D_s=50$, $\tau =0.01$s, $\omega _s=100\pi /s$. $T=2.0$s in (**A**), (**B**,**D**) and $T=400.0$s in (**C**) for visualisation reasons.

A potential explanation of this phenomenon is found by studying the effects of such stimuli on individual uncoupled cells. In Fig. 7 these effects were quantified via the power spectral density for an individual modeled excitatory and inhibitory cell; the underlying mathematical derivations for these curves can be found in “Methods” section. For all four stimulus paradigms, inhibitory cells respond more strongly than excitatory cells (shown by the larger power spectral densities) for all frequencies. We note that the stimulation paradigms in Fig. 7A,B yield several clear spectral peaks at equidistant frequencies, which are multiples of the pulse repetition frequency and their power-spectral density is proportional to the repetition frequency. The sinusoidal stimulation paradigm presented in Fig. 7C yields a single spectral peak with a low magnitude, since this magnitude is not frequency dependent (see “Methods” section for more details). The noise stimulation paradigm in Fig. 7D does not exhibit any spectral peak since the input is white noise whose frequencies have identical magnitude.

As derived in “Methods” section, these differential responses at the single cell level can be explained by the different time scales of the excitatory and inhibitory cells, which represent experimentally observed differences in the membrane time constants of cortical pyramidal neurons and parvalbumin (PV) positive interneurons^61^. Taken together, these results indicate that these differential time scales promote an enhanced response of inhibitory cells over excitatory cells to the type of stimuli studied here, in turn potentially explaining the finding in Fig. 6 that, at high enough stimulus amplitudes, inhibitory cells become more active than excitatory cells.

## Discussion

In this study, we use a variety of computational techniques, including numerical simulation and mean-field mathematical analysis, to probe the influence of both intrinsic noise and extrinsic stimulation (both noisy and periodic) on the stability of a spiking neural network. Our results show that both intrinsic and extrinsic noisy inputs stabilize our microcircuit, making it less susceptible to the sudden transition into oscillatory dynamics that is often used to model seizure onset. Building on previous results^57,58^, we have extended these findings to other periodic stimuli and hence gleaned a better understanding of the relationship between sudden transitions into oscillatory activity and neuromodulation using a mathematical and computational lens.

Our analysis shows that noise serves to suppress multi-stable behavior, leading the system into a regime in which the network’s response to changing input is commensurate to the magnitude of that change. Furthermore, noise minimizes the activity of excitatory cells in these driven states (see Figs. 2, 3) by repositioning the system’s equilibrium point. Simulations in the spiking model show that various combinations of stimulation waveforms, frequencies and amplitudes can stabilize network activity in a similar manner, with the coherence following an increase in external drive (the seizure stimulus) typically minimized for high-frequency and high-amplitude stimulation. This regime of minimal coherence represents the stimulation paradigms with anti-ictogenic effects, and effective paradigms with minimal frequency or amplitude can be determined by analyzing Fig. 5. Detailed analysis of the activity of the excitatory and inhibitory populations further reveal a selective and predominant recruitment of inhibitory cells: more strongly driven by high-frequency inputs, the inhibitory population suppresses excitatory activity and biases the system towards a desynchronized state antithetical to seizure.

The models used here are chosen purposefully to retain both mathematical tractability and the ability to draw parallels between related population and spiking models. The Wilson–Cowan equations^53^ are a frequently used formulation in this endeavor. These choices allow us to simultaneously consider three key points in our in silico investigations: first, the correspondence between mathematical analysis of the population model and the simulated behavior of the related spiking model (we note the correspondence of the predictions of our analyses of these population models with the computational results of the related spiking model); second, the differential role of excitatory and inhibitory cell populations in seizure onset in both settings; and third, the effects of neuromodulatory intervention on both population-level and cell-level dynamics related to seizure onset, using both mathematical and computational tools. In this manner, we have provided theoretical and mathematical support for our hypotheses regarding the relationships between intrinsic noise, neuromodulatory paradigms, and seizure onset. Work using more detailed neural models, potentially including the implementation of features such as spike timing dependent plasticity (STDP), is a fertile ground for future research to probe these hypotheses in a more biophysically constrained setting. Additional future work could investigate how in vitro neuromodulatory stimuli can be optimized so as not to induce oscillatory activity, with our analysis here providing initial evidence that stimulation with a sufficiently high frequency may accomplish this through selectively activating inhibitory cells in a non-rhythmic fashion. The use of bio-hybrid systems^92–95^ could provide an useful tool in such research.

A paramount use of these mathematical findings is towards the articulation of a theoretical understanding of how neuromodulation acts to mitigate seizure onset, and in turn potential pathways through which these treatments might be improved. While the oscillatory dynamics observed in our simulations are not meant as “model seizures” (indeed, there are crucial features that differentiate seizures from general oscillatory dynamics, such as the “glissandi”^96^), fully encapsulating all of these features is outside the realm of this study considering the desire to preserve the mathematical tractability of this model. Instead, the conclusions drawn from this work remain focused on seizure *onset*. This focus is motivated by the similarities between the transitions between asynchronous and oscillatory dynamics in this model and important features of the well-described low-voltage fast-onset transition into seizure. These include maintaining “intact inhibition” during this transition^82,83^ and the initial oscillatory frequencies in the beta/gamma range^80–82^. Considering the focus on seizure onset, this model can be viewed as analogous to a mesoscopic population making up a few millimeters of tissue, given experimental evidence that ictal states emerge in brain regions of this scale^55,97–99^.

The critical anti-ictogenic role of inhibitory interneurons is well established in the epilepsy literature^96,100^, with many anti-seizure interventions aiming to provide additional inhibition via the activation of inhibitory receptors^101,102^ or directly rescuing the activity of inhibitory interneurons^103,104^. Neuromodulation has been shown to be an effective treatment for many individuals that do not respond to these treatments^3–7^, with the commonly hypothesized mechanism of action for analogous DBS systems being the creation of a “functional” lesion, potentially via depolarization blockade^16,19^. Our results present theoretical support for a distinct mechanism of action for the effects of neuromodulation via the upregulation of inhibitory activity. Simultaneously, these findings highlight a very important practical aspect of clinical neuromodulation which is that neuromodulatory devices need not be overly precise (i.e. selectively target only inhibitory cells), given the different responses observed in excitatory and inhibitory cells to global stimulation in our in silico explorations.

However, the seemingly paradoxical role of interneurons in seizure must be taken into account when interpreting these findings. While interneurons serve a clear role in restraining seizure propagation, there is also evidence that interneurons are complicit in seizure initiation, potentially serving a causal role^38,43,44,105–108^. One hypothesized pathway through which interneuron hyperactivity may contribute to seizure is through the accumulation of chloride in these circuits which can make GABAergic signalling excitatory instead of inhibitory^37,109^, a behavior not implemented in our model to preserve mathematical tractability. Nonetheless, when considering neuromodulatory paradigms in the in vivo setting these factors must still be considered.

Thus, it is likely that in vivo neurostimulation paradigms motivated by this work would have to be temporally limited to avoid potentially initiating, rather than suppressing, seizure by overstimulating inhibitory cells. Indeed, while neuromodulation is a relatively recent clinical treatment to prevent seizure, it has long been known to have ictogenic potential^110^. Our results may correspondingly help design stimulation paradigms that increase interneuronal firing intermittently and transiently in order to avoid hyperactivity in interneurons and preserve their stabilizing effects. In this vein, our previous work explored how a hyperexcitable purely interneuronal network is more likely to serve as a potential “trigger” starting a seizure than a less excitable interneuronal network^45^. Together, our findings suggest that neuromodulatory strategies will need to be tuned to avoid the pro-ictogenic effects of excessively increasing interneuronal activity (potentially by triggering a non-linear state transition amongst interneuronal networks), while exploiting the anti-ictogenic effects we describe here.

We note that our goal here was not to explore our entire model parameter space, but instead provide an example of how a specific type of state transition can be suppressed by neurostimulation and what potential mechanisms underlie this phenomenon. While various bifurcations scenarios can lead to seizure-like dynamics^26,111^, we argue that the waveforms explored here can stabilize neural activity and thus provide a generic mechanism to prevent multi-stability and other types of non-linear properties arising in a variety of neural networks.

Despite the theoretical nature of this work and its corresponding neuroscientific applications, there are a plethora of known similar noise-induced effects in physical systems that can be seen as support for the feasibility of translating these in silico findings to the physical brain. The generic mechanism by which additive noise stabilizes the dynamics of non-linear systems is seen in systems with threshold-like non-linearities such as digital audio, image processing, optics, communications systems and stock market fluctuations (see^112^ and related literature on dithering). In neuroscience specifically, this occurs in individual neurons^113^, delayed-feedback systems^56,79^ and plays a role in stochastic^87^- and coherence-resonance^114^, which are widespread in across physical and neural systems.

Furthermore, there are existing experimental examples within the field of neuroscience where the theoretical mechanisms explored here have been practically implemented in order to suppress pathological dynamics. Sensory stimulation has long been shown to halt or shorten seizure-like events^31,115^, and it makes intuitive sense that techniques to stop an existing seizure may have parallels to techniques to prevent the onset of seizure altogether. Other types of stimuli, such as vagus nerve stimulation, activate upstream neural populations, which translates as an increased afference of spiking inputs at the epileptic focus^10^. While our simplistic model is far from capturing the full complexity of the mechanisms involved in the examples above, our analysis provides theoretical support for the hypothesis that noise, either intrinsic or extrinsic, serves to stabilize the dynamics of neural networks in a way may oppose seizure initiation, and articulates pathways through which the effects of these extrinsic inputs can be optimized.

## Supplementary information

Supplementary information.

## Data Availability

The code used in this study will be made available upon request to the corresponding author.
