# Supplementary material for: Neurostimulation stabilizes spiking neural networks by disrupting seizure-like oscillatory transitions
Source: Sci Rep. 2020 Sep 21;10:15408. doi: 10.1038/s41598-020-72335-6 (PMC7506027; doi:10.1038/s41598-020-72335-6)
Supplement: Supplementary file 1 — Supplementary information. [file 41598_2020_72335_MOESM1_ESM.pdf]

Neurostimulation stabilizes spiking neural networks by disrupting seizure-like oscillatory transitions

Scott Rich

Krembil Research Institute, Division of Clinical and Computational Neuroscience,
Toronto, Ontario, Canada

Axel Hutt

Team MIMESIS,
INRIA Nancy Grand Est, Strasbourg, France

Frances K. Skinner

University of Toronto, Departments of Medicine (Neurology) and Physiology,
Toronto, Ontario, Canada

Taufik A. Valiante

University of Toronto, Institute of Biomaterials and Biomedical Engineering,
Toronto, Ontario, Canada
University of Toronto, Institute of Medical Science,
Toronto, Ontario, Canada
University of Toronto, Department of Surgery, Division of Neurosurgery,
Toronto, Ontario, Canada
University of Toronto, Electrical and Computer Engineering,
Toronto, Ontario, Canada

Jeremie Lefebvre

Krembil Research Institute, Division of Clinical and Computational Neuroscience,
Toronto, Ontario, Canada
University of Ottawa, Department of Biology,
Ottawa, Ontario, Canada
University of Toronto, Department of Mathematics,
Toronto, Ontario, Canada

Appendix

The mean field equation reads

$$\begin{aligned} a_e^{-1}\frac{d}{dt}\bar{u}^e &= L\left[\bar{u}^e\right] + \bar{w}^{ee}\ F_e\left[\bar{u}^e\right] + \bar{w}^{ie}F_i\left[\bar{u}^i\right] + I^e + I^o + \mu_S \\ a_i^{-1}\ \frac{d}{dt}\bar{u}^i &= L\left[\bar{u}^i\right] + \bar{w}^{ei}\ F_e\left[\bar{u}^e\right] + \bar{w}^{ii}F_i\left[\bar{u}^i\right] + I^i + \mu_S \end{aligned} \tag{1}$$

where $\bar{w}^{nm}$ denotes the synaptic weight from population m to population n. One may define the level of excitatory and inhibitory interaction by $E = \bar{w}^{ee} + \bar{w}^{ei}$ and $I = \bar{w}^{ie} + \bar{w}^{ii}$, respectively. Consequently the E/I balance is defined by the ratio E/I.
To investigate how the system dynamics change with different E/I values, we define E_0 and I_0 by the values given in Table 1 and keep $\bar{w}^{ie}$ and $\bar{w}^{ii}$ constant . Then we choose

$$\bar{w}^{ee} = \frac{E/I}{E_0/I_0}\bar{w}_o^{ee}\ ,\ \bar{w}^{ei} = \frac{E/I}{E_0/I_0}\bar{w}_o^{ei}$$

where $\bar{w}_o^{ee}$ and $\bar{w}_o^{ei}$ are chosen to the values in Table 1. We note that $E_0/I_0 = 0.95$, i.e. excitatory and inhibitory interactions are almost balanced in Figures 1-7 in the main manuscript.

Supplemantary Figure S1 presents the equilibrium $\bar{u}_0^e$, for which $d\bar{u}^e/dt = 0$, $d\bar{u}^i/dt = 0$, dependent on the excitatory input I_0 for different values of E/I. For the low noise level $D = 0.005$, we observe multi-stability states between $2.85 \geq E/I \geq 0.29$, whereas the low value $E/I = 0.08$ yields a single equilibrium in that range of I_0. For larger noise level $D = 0.05$, no multi-stable states occur anymore and for $2.85 \geq E/I \geq 0.29$ the equilibria are stable. Consequently the results in the main text for $E_0/I_0 = 0.95$ are robust towards changes in the E/I balance.

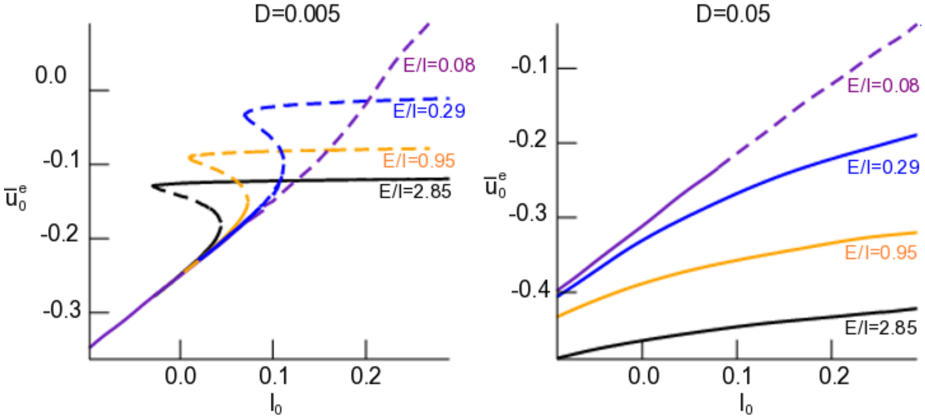

Supplemantary Figure S1: **Effect of E/I-balance on stabilization by stochastic stimulation**. Increasing the constant input I_0 destabilizes the equilibrium state $\bar{u}_0^e$. Left panel: low noise variance $D = 0.005$. Right panel: large noise variance $D = 0.05$. In both panels, solid lines denote asymptotically state equilibria and dashed lines mark asymptotically unstable equilibria.
